# hERG Potassium Channel Blockage by Scorpion Toxin BmKKx2 Enhances Erythroid Differentiation of Human Leukemia Cells K562

**DOI:** 10.1371/journal.pone.0084903

**Published:** 2013-12-26

**Authors:** Jian Ma, Youtian Hu, Mingxiong Guo, Zan Huang, Wenxin Li, Yingliang Wu

**Affiliations:** 1 State Key Laboratory of Virology, College of Life Sciences, Wuhan University, Wuhan, China; 2 Department of Biochemistry and Molecular Biology, College of Life Sciences, Wuhan University, Wuhan, China; Institut national de la santé et de la recherche médicale - Institut Cochin, France

## Abstract

**Background:**

The hERG potassium channel can modulate the proliferation of the chronic myelogenous leukemic K562 cells, and its role in the erythroid differentiation of K562 cells still remains unclear.

**Principal Findings:**

The hERG potassium channel blockage by a new 36-residue scorpion toxin BmKKx2, a potent hERG channel blocker with IC_50_ of 6.7±1.7 nM, enhanced the erythroid differentiation of K562 cells. The mean values of GPA (CD235a) fluorescence intensity in the group of K562 cells pretreated by the toxin for 24 h and followed by cytosine arabinoside (Ara-C) treatment for 72 h were about 2-fold stronger than those of K562 cells induced by Ara-C alone. Such unique role of hERG potassium channel was also supported by the evidence that the effect of the toxin BmKKx2 on cell differentiation was nullified in hERG-deficient cell lines. During the K562 cell differentiation, BmKKx2 could also suppress the expression of hERG channels at both mRNA and protein levels. Besides the function of differentiation enhancement, BmKKx2 was also found to promote the differentiation-dependent apoptosis during the differentiation process of K562 cells. In addition, the blockage of hERG potassium channel by toxin BmKKx2 was able to decrease the intracellular Ca^2+^ concentration during the K562 cell differentiation, providing an insight into the mechanism of hERG potassium channel regulating this cellular process.

**Conclusions/Significance:**

Our results revealed scorpion toxin BmKKx2 could enhance the erythroid differentiation of leukemic K562 cells via inhibiting hERG potassium channel currents. These findings would not only accelerate the functional research of hERG channel in different leukemic cells, but also present the prospects of natural scorpion toxins as anti-leukemic drugs.

## Introduction

Human erythropoiesis is a complex multi-step developmental process that begins at the level of hematopoietic stem cells (HSCs) at bone marrow microenvironment and terminates with the production of erythrocytes. Erythropoiesis is one of the most important physiological activity for human, around 2×10^11^ erythrocytes must be replaced each day to maintain adult human haemopoiesis [1]. So far, it is well known that erythropoiesis is regulated at various levels by microenvironmental, transcription factors(GATA-1, FOG-1, PU-1, etc.), micro-RNAs and many signaling pathways (HIF, EpoR, Wnt, etc.) [2]. Interestingly, human potassium channels, as the diverse and ubiquitous membrane proteins, serve a variety of physiological and pharmacological functions [3-6], and they were also found in the different normal or neoplastic cells during the hematopoietic process. In the hematopoietic stem cells, RT-PCR of potassium channel mRNAs indicated the coexperession of Kv1.3 and Kv7.1 potassium channels [7]. The hERG (human ether-a-go-go-related gene) potassium channels were also expressed in a variety of cancer cells whereas the corresponding non-cancerous cells and cell lines had no significant hERG protein expression. In particular, overexpression of hERG channels was observed in various types of neoplastic hematopoietic cells. For example, the hERG channels were found to be expressed in different leukemic cells, such as CEM, K562 and U937 [8], and the expression of hERG channels was also detected in the stimulated CD34^+^ cells of leukemic patients [9]. Pharmacological experiments showed that the hERG channels could be blocked by the chemical molecule blockers of E-4031 or Way123,398. Functionally, the blockage of hERG channels by the chemical molecules was found to be able to inhibit the proliferation of leukemic cells [8-10]. Due to the complexity of erythropoiesis and other hematopoietic process, more functions of the potassium channels remain unclear so far.

The functions of potassium channels are usually explored by their specific animal toxin blockers. Scorpion toxins are known peptide blockers interacting with the extracellular pore entryway of the different potassium channels, whose inhibitory mechanism is different from that of the chemical molecule blockers [11-16]. Structurally, these toxins typically contain about 30-40 amino acid residues with 3-4 disulfide bridges usually linking an helix and two- or three-stranded β-sheet structures [13]. At present, these scorpion toxins are extremely useful molecular tools to probe the structure-function information of potassium channels [16,17], and become valuable resources of peptide drug discovery [6,18]. In this work, a hERG potassium channel sensitive scorpion toxin, the 36-residue BmKKx2 peptide [19], was used to investigate the effect of hERG channel on the erythroid differentiation of human leukemia cells K562. It was found that scorpion toxin BmKKx2 was able to reduce the proliferation and enhance the erythroid differentiation of K562 cells through interacting with hERG potassium channel. Furthermore, the specific blockage of scorpion toxin BmKKx2 could suppress the expression of hERG potassium channel and decrease the Ca^2+^ concentration during the erythroid differentiation of K562 cells. Together, these findings not only illustrated the novel function of hERG potassium channel during the erythroid differentiation of the leukemia cells, but also presented the potential application of scorpion toxins as anti-leukemic drugs.

## Materials and Methods

### Cell counting and MTT assay

The effect of hERG blockage via toxin BmKKx2 on K562 cell proliferation was tested by the MTT assay and cell counting. The K562 cells (CCTCC, Wuhan, China) were cultured in RPMI1640 medium containing 10% (v/v) fetal bovine serum (GIBCO), penicillin (100 U/ml), and streptomycin (100 mg/ml). Cells were maintained at 37°C in a 5% CO_2_ incubator. 

For MTT assay, the K562 cells (1×10^5^ cells/ml) were seeded in triplicate in RPMI1640 medium in 96-microwell plates (Nest) and were incubated with or without BmKKx2 (200nM) for 48 hours. After 10 μl of a 5 mg/ml stock solution of MTT (3-[4, 5-dimethylthiazol-2, 5-diphenyl tetrazolium bromide]) (Sigma) was added to each well for the last 4 hours of the culture, the formed formazan crystals were solubilized by adding 150 μl DMSO. Then the plates were measured for optical density by a dual-beam microplate reader (BioTek) using a test wavelength of 570 nm with a reference of 630 nm. For cell counting, 5×10^4^ cells were cultured in triplicate in 24-well plates. Cells were counted in a hemocytometer every other day. After 72 h, cells were removed to 6-well plates and half of the media were renewed daily.

### Erythroid differentiation of K562 cells

The erthyroid differentiation of K562 cells were induced by anti-metabolite cytosine arabinoside (Ara-C) in a final concentration of 1 μM [20]. BmKKx2 is diluted in 0.5% (w/v) BSA solution to a final concentration of 200 nM. K562 cells treated for 24 h, 48 h, 72 h were collected to investigate the differentiating process.

Expression of the erythroid-specific surface marker glycophorin A (GPA, also named as CD235a) was tested by Flow Cytometry to confirm the differentiation. Briefly, 1×10^6^ cells were incubated at 4°C in the dark for 30 minutes with 100 μl diluted fluorochrome-conjugated GPA antibody (BD) at a final concentration of 10 μg/mL. Then the cells were washed twice with ice-cold PBS (pH 7.4) to remove unbound antibody and resuspended in 300 μl cold PBS (pH 7.4) for FCM (Beckman). Ten thousand events were analyzed for each sample by FACScan.

### Cell lysis and Western blotting

Cells were lysed with RIPA buffer (50 mM Tris-HCl, pH 7.5; 150 mM NaCl; 1% (v/v) NP-40; 0.25% (w/v) sodium deoxycholate). Equal amounts of extract were then electrophoresed on a 10% (w/v) sodium dodecyl sulfate polyacrylamide gel electrophoresis gel (SDS-Page), transferred to nitrocellulose filter membranes (Millipore) and immunoblotted with appropriate antibodies. Anti-hERG antibody (1:500) and anti-γ-globin (1:1000) were bought from Abgent, and anti-HSC70 (1:2000, Proteintech) was used as the endogenous control. For detection, they were then incubated with the horseradish peroxidase-conjugated secondary antibodies (Pierce), and immunoreactivity was visualized using the SuperSignal chemiluminescent detection module (Pierce).

### Electrophysiological recording

K562 cells were incubated in the external solution consisted of (in mM): 137 NaCl, 4 KCl, 1 MgCl_2_, 1.8 CaCl_2_, 10 D-Glucose and 10 HEPES (pH 7.4 with NaOH); and the internal solution contained (in mM): 130 KCl, 1 MgCl_2_, 5 MgATP, 5 EGTA, 10 HEPES (pH 7.2 with KOH). K562 cells expressing hERG channel were depolarized from a holding potential of –80 mV to +40 mV for 500 ms then hyperpolarized to –120 mV for 1 s and current amplitudes were measured from the peak inward current at –120 mV. Membrane currents were measured with an EPC 10 patch clamp amplifier (HEKA Elekt-ronik, Lambrecht, Germany) interfaced to a computer running acquisition and analysis software (Pulse).

BmKKx2 was prepared as a 50 mM stock solution in 0.5% BSA, stored at -20 °C, and then diluted in bath solution to the final concentration. BmKKx2 was expressed by *E. coli* Rosetta (DE3) cells according to previous techniques of our group [19].

### Real-time quantitative PCR

For all cells and cell lines the total RNA was extracted using the TRIzol method (Invitrogen) and total RNA was reverse transcribed into cDNA using SuperScript II (Invitrogen, Carlsbad, CA, USA). Real-time quantitative PCR was performed using an ABI 7500 real-time PCR system (Applied Biosystems, Foster City, CA, USA) and the SYBR Green Real time PCR Mater Mix (TOYOBO, Osaka, Japan). Primers sequences used for real-time quantitative PCR are obtained from previous study [21]. Each PCR reaction was performed in triplex tubes, with glyceraldehyde 3-phosphate dehydrogenase (GAPDH) being used as an endogenous control to standardize the amount of sample RNA.

### Ca^2+^ concentration recording

Free Ca^2+^ concentration was detected by Flow Cytometry with the Fluo-8/AM (AAT Bioquest). Cells were loaded with Fluo-8/AM at 37°C in the dark at a final concentration 5 μM in complete culture medium. After 30 min incubation, the cells were washed twice to remove excess probes and resuspended in 500 μl PBS. Ten thousand events were analyzed for each sample by FACScan.

### Generation of hERG-deficient cell lines

Lentiviral particles containing short hairpin RNA (shRNA) targeted to hERG mRNA and its control vector were establish by vector pLKO.1-puro. Three shh-hERG lentiviral particles were designed and vector with scrambled shRNA was used as control. K562 cells were transfected with hERG shRNA lentiviral particles (shh-hERG) or shRNA particles containing vain plasmid (shh-control). The shRNA (shh-hERG) sequences used were as follows: shh-1 5’-CCG GCC TGC GAG ATA CCA ACA TGA TCT CGA GAT CAT GTT GGT ATC TCG CAG GTT TTT G-3’; shh-2 5’-CCG GCC GTA AGT TCA TCA TCG CCA ACT CGA GTT GGC GAT GAT GAA CTT ACG GTT TTT G-3’; shh-3 5’-CCG GCC CTC CAT CAA GGA CAA GTA TCT CGA GAT ACT TGT CCT TGA TGG AGG GTT TTT G-3’. K562 cells were cultured in media containing recombinant lentiviral particles and 1 mg/ml polybrene for at least 48 h and then moved to puromysin selection for one week. The survival cells were stably silenced hERG K562 cells.

### The cell cycle profile and annexin V/propidium iodide (PI) staining

The cell cycle profile was analyzed by treating cells with 70% (v/v) ethanol overnight at 4 °C and staining them with PI. Apoptosis was measured by staining cells with PI and FITC-conjugated Annexin V (MultiSciences Biotech). Cells were cultured in the indicated scheme for the specified time, collected by centrifugation, and washed with PBS. Cells were stained with annexin V-FITC and PI and incubated for 10 min at room temperature in the dark. Ten thousand events were analyzed for each sample by FACScan. FACS analysis was performed using Flowjo.

## Results

### Scorpion toxin BmKKx2 blocking hERG channel currents in K562 cells

**Figure 1:**
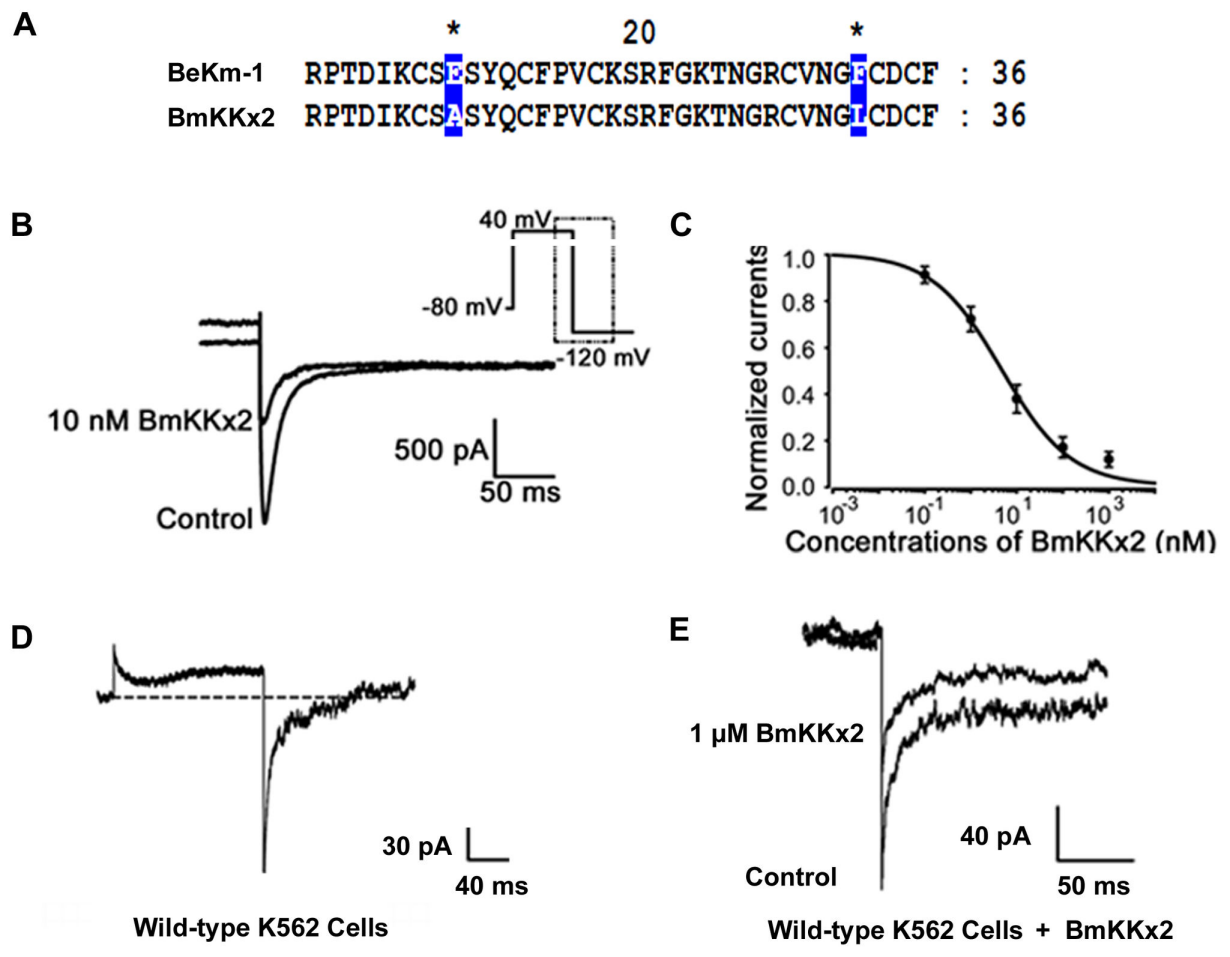
Scorpion toxin BmKKx2 primary structure and its pharmacological effect on the hERG channel. (A) the sequence alignments between scorpion toxins BmKKx2 and BeKm-1 [11,19]. (B) The pharmacological effect of BmKKx2 on hERG channels. The hERG channels were transfected in HEK 293 cells, and their current traces were shown in the absence (control) or presence 10 nM BmKKx2. (C) Dose-dependence curve of BmKKx2 on hERG channels expressed in HEK 293 cells. Symbols and associated error bars represent means ± SD for several cells (n=5). (D) The current trace of hERG potassium channels in absence of BmKKx2 in wild type K562 cells; (E) The pharmacological effect of BmKKx2 on hERG channels in wild type K562 cells. Current traces were shown in the absence (control) or presence 1 μM BmKKx2 .

Scorpion toxin BmKKx2 shares the similar structure to scorpion toxin BeKm-1, a known hERG channel-selective blocker with an IC_50_ value of 3.3 nM [11,19,22] (Figure **1A**), which suggests that BmKKx2 may also act as a hERG channel blocker. As expected, 10 nM recombinant BmKKx2 could remarkably block ~60% currents of hERG channels, which were transiently expressed in HEK 293 cells (Figure **1B**). The concentration-dependent experiments further indicated the recombinant BmKKx2 could block hERG channel with an IC_50_ of 6.7±1.7 nM (Figure **1C**). 

Next, we investigated whether scorpion toxin BmKKx2 could inhibit the hERG channel currents in K562 cells, a chronic myelogenous leukemic cell line experessing hERG potassium channels [8-10]. In this work, the hERG channels were activated at the holding potential of +40 mV, and the inward tail currents of hERG channels were measured. As shown in Figure **1D**, typical hERG channel currents were detected in wild-type K562 cells, and about 50% currents were blocked in the presence of 1 μM BmKKx2 (Figure **1E**). This data showed that the scorpion toxin BmKKx2 could moderately inhibit hERG channel currents in K562 cells, which is different from the nearly complete inhibition of hERG channel currents by the chemical molecules [8-10]. Since both the full-length and N-terminally truncated isoform of hERG channel were found to be co-expressed in K562 cells [8-10], and these homotetrameric and heterotetrameric channels would likely affect the inhibitory effect of toxin BmKKx2. Such differential pharmacological effects were observed between the scorpion toxin charybdotoxin and chemical molecules acting on the full-length and N-terminally truncated isoform of Kv1.3 channel, respectively [23].

### hERG channel blockage by toxin BmKKx2 suppressing proliferation and enhancing erythroid differentiation of K562 cells

**Figure 2:**
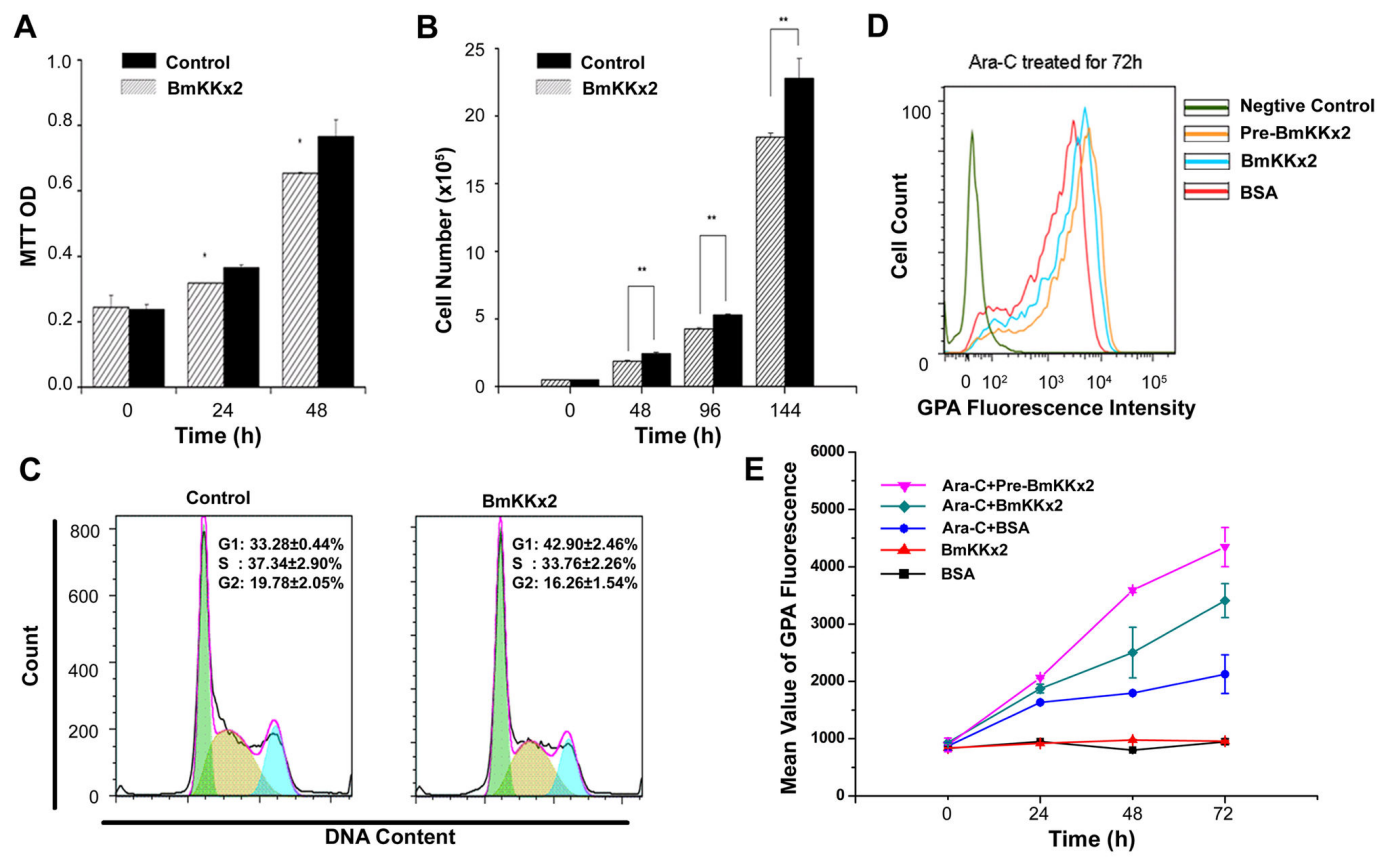
BmKKx2 suppressing proliferation and enhancing erythroid differentiation of K562 cells. (A) Cellular proliferation determined by MTT assay for different time. (B) Cellular proliferation determined by cell counting for different time. Values are derived from an average of three independent experiments, error bars represent means ± SD for three replicates. *p<0.05 and **p<0.01 (Student’s *t*-test). (C) Cell cycle profiles assessed by DNA content in PI-stained cells. The proportion of cell in different phase was calculated, and the values from three independent experiments were expressed as mean ± SD. (D) GPA fluorescence intensity in cells treated Ara-C for 72 h as measured by the flow cytometry. For Pre-BmKKx2 goup, BmKKx2 was added 24 h before Ara-C treatment; for BmKKx2 group, BmKKx2 and Ara-C were added at the same time; for BSA group, only Ara-C was added. PE-conjugated IgG was served as the negative control. (E) GPA expression in different induce conditions shown by mean values of fluorescence intensity. Values represent the means ± SD for the separate determinations.

As a novel peptide blocker of hERG channel, the role of scorpion toxin BmKKx2 was investigated in the proliferation of K562 cells. As shown by the MTT assay, toxin BmKKx2 could decrease the number of K562 cells at 48 h compared with control wells by ~17% (Figure **2A**), and the similar effect of BmKKx2 on K562 cell proliferation was also illustrated by the cell counting experiments. The number of cells treated with 200 nM BmKKx2 was approximately 20% lower than those of control group at 144 h (Figure **2B**). The FACS analysis of PI-stained cells further revealed that the proportion of cells in S phase was decreased by about 3.6% and the proportion of cells in G1 phase increased by approximately 9.6% in the presence of 200 nM BmKKx2, indicating BmKKx2 could suppress the cell growth by inducing a specific accumulation at G1 phase of the cell cycle (Figure **2C**). All these data proved that BmKKx2 could decelerate the proliferation of K562 cells, which was similar to the effect of chemical molecule blocker E-4031 [8].

The most important feature of leukemia cells is malignant proliferation and loss of the ability to differentiate. When scorpion toxin BmKKx2 reduced the proliferation of K562 cells, it was beneficial to investigate whether BmKKx2 would enhance erythroid differentiation of K562 cells by inhibiting the hERG potassium channel. In this work, the K562 cell differentiation induced by Ara-C [20] was found to be enhanced by adding 200 nM BmKKx2 into K562 cells, which was illustrated by the higher GPA (CD235a) fluorescence intensity (Figure **2D**) and bigger mean value of GPA intensity (Figure **2E**). At 72 h, the mean value of GPA intensity in the group of K562 cells treated by toxin BmKKx2 was about 1.6-fold stronger than that of Ara-C alone group (Figure **2E**). Interestingly, the erythroid differentiation was further accelerated when K562 cells were pretreated with 200 nM BmKKx2 for 24 h before adding Ara-C (Figure **2D**), and the mean value of GPA intensity was about 2-fold stronger than that of Ara-C induced alone cells (Figure **2E**). Based on the more significant effect of the pretreated BmKKx2, the toxin dose-dependent experiments were further conducted. As shown in Figure **S1**, the K562 cell differentiation was continuously enhanced with the increasing BmKKx2 concentration. Considering the balance between toxin efficiency and amount, 200 nM BmKKx2 was selected with the pre-incubation strategy in the following work. In addition, the similar effect of BmKKx2 on the cell differentiation was also observed in the hemin-induced K562 cells (Figure **S2**).

Together, all these results indicated the novel function of scorpion toxin BmKKx2 in suppressing proliferation and enhancing erythroid differentiation of K562 cells.

### Scorpion toxin BmKKx2 had no effect on the erythroid differentiation of K562 cells after hERG channel knockdown

**Figure 3:**
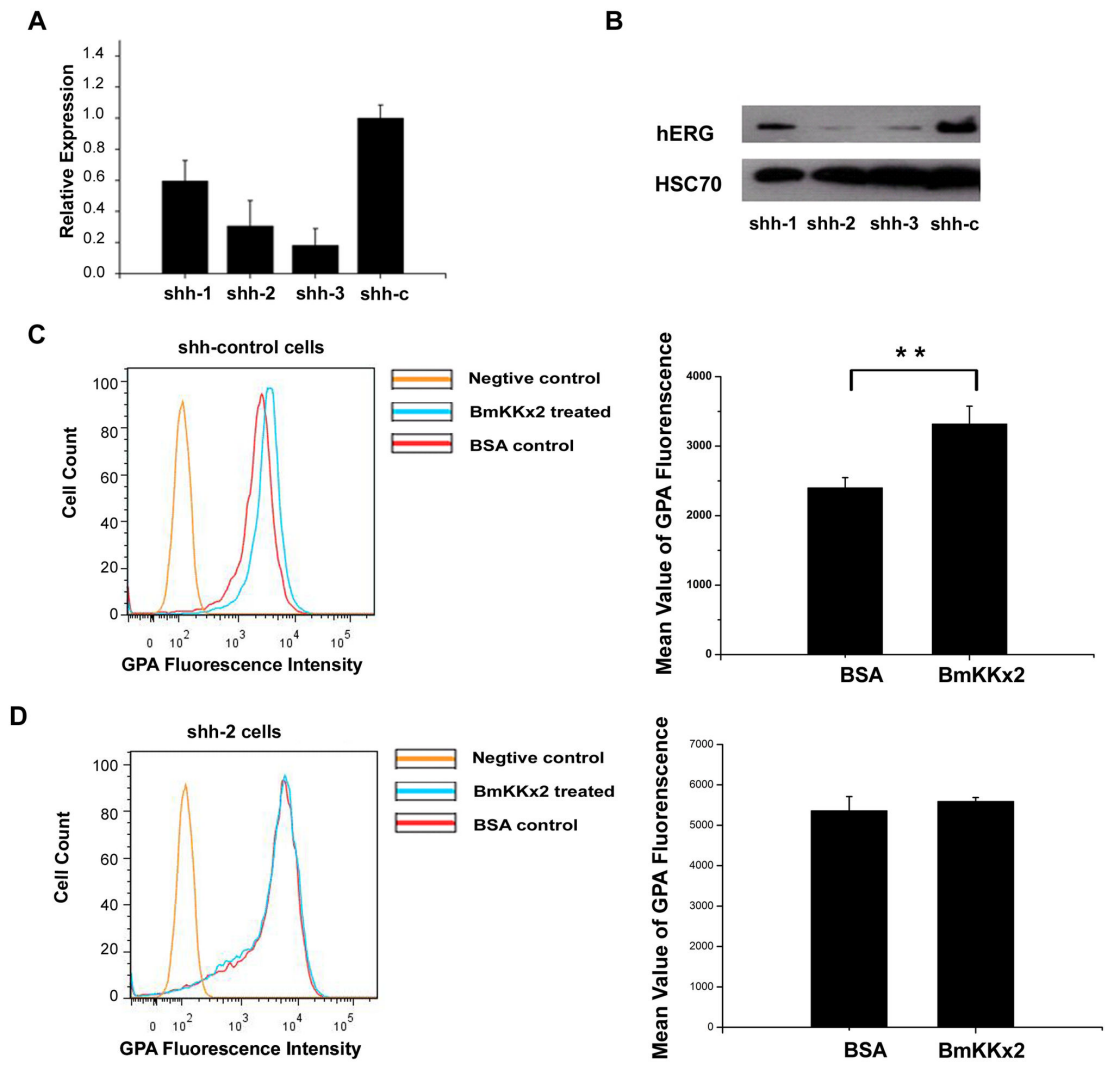
No effect of BmKKx2 on the erythroid differentiation in the hERG channel-deficient K562 cells. (A) hERG channel expression in hERG-silenced and control cells shown by quantitative real-time PCR. (B) hERG channel expression shown by western blotting analysis. HSC70 was used as the endogenous control. (C) BmKKx2 enhancing the differentiation in the lentiviral vector-infected control cells. (D) BmKKx2 with no effect on the differentiation of the hERG-silenced cells. K562 cells in (C) and (D) were treated with Ara-C for 48 h. The right panel of (C) and (D) showed the mean values of GPA fluorescence from three independent samples. **p<0.01 (Student’s *t*-test).

To confirm whether scorpion toxin BmKKx2 affected the differentiation of K562 cells through interacting with hERG channel, the strategy of hERG potassium channel knockdown was used in this work. By using the recombinant lentiviral particles containing hERG shRNAs, we established hERG channel-deficient cell lines and the differential expression of hERG potassium channel was shown at the mRNA and protein levels (Figure **3A and 3B**). The lowest expression of hERG channel was found in K562 cells transfected by the shh-2 particle, which was used to investigate the role of hERG channel in the differentiation of K562 cells.

Next, the hERG channel-deficient cell lines were then treated by Ara-C with or without toxin BmKKx2 as described previously. As shown in Figure **3C**, the control cell line still showed its sensitivity to BmKKx2 with increasing GPA fluorescence intensity due to the higher expression of hERG potassium channels. However, the GPA fluorescence intensity had no obvious difference between the BmKKx2 treated and untreated groups when the expression of hERG potassium channel was suppressed (Figure **3A, 3B** ****and **3D**). Those results further revealed that toxin BmKKx2 was able to accelerate the K562 cell differentiation through interacting with hERG channels.

### BmKKx2 suppressed the expression of hERG channel during the erythroid differentiation of K562 cells

**Figure 4:**
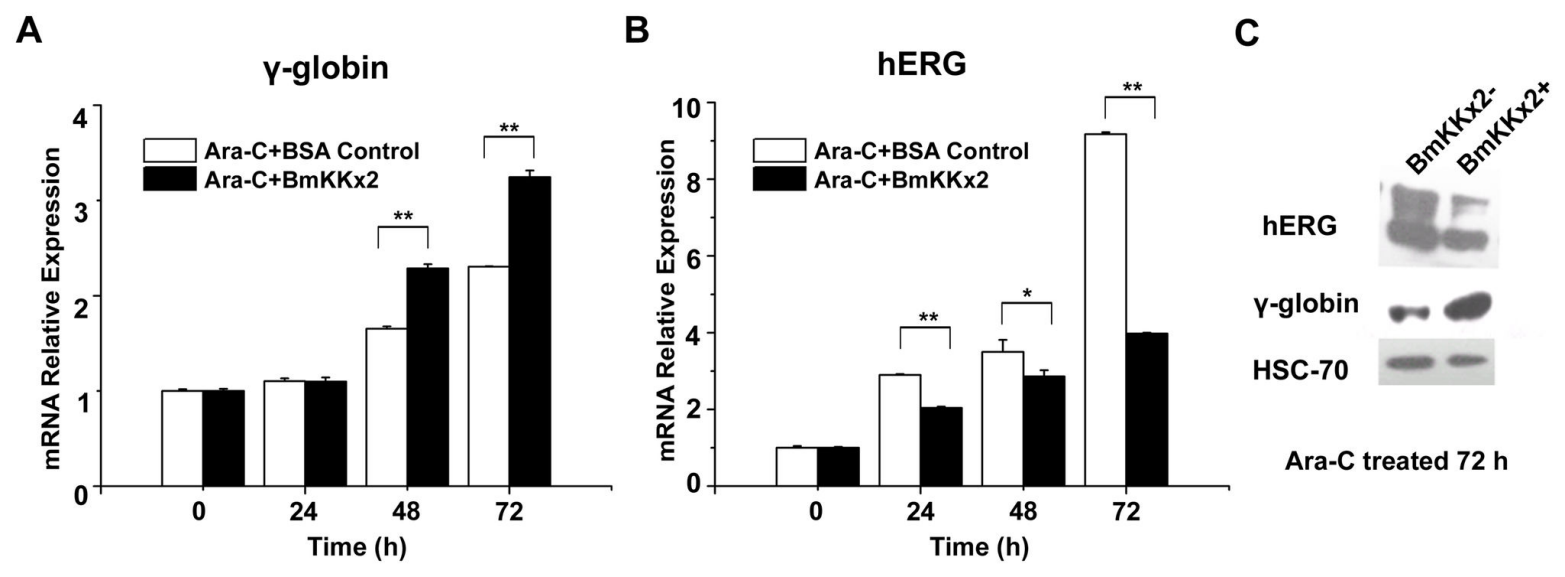
hERG channel expression suppressed by BmKKx2 in erythroid differentiation of K562 cells. (A) γ-globin expression in K562 cells during the differentiation with or without BmKKx2 shown by quantitative real-time PCR. (B) hERG channel expression in K562 cells during the differentiation process with or without BmKKx2 shown by quantitative real-time PCR. **p<0.01 (Student’s *t*-test). Symbols and associated error bars represent means ± SD for three independent experiments (C) hERG and γ-globin expression in K562 cells with or without BmKKx2 tested by the western blotting analysis. Both HERG1 and HERG1B isoforms were detected [24].

During the process of K562 differentiation, the progenitor cells change into highly specialized cells making large quantities of hemoglobin, and a large mount of membrane proteins were likely regulated in this process. In this work, the expression of hERG channel, a novel differentiation accelerating factor for K562 cells, was also investigated in the absence and presence of scorpion toxin BmKKx2. As shown in Figure **4A**, the quantitative real-time PCR showed the up-regulation of the γ-globin, an indicator for the erythroid differentiation of K562 cells. In line with previous data of BmKKx2 enhancing the differentiation of K562 cells, the γ-globin experession was more significant in the BmKKx2 treated cells than that of the BmKKx2 untreated cells (*p*<0.01). Meanwhile, the hERG channel expression was also increased along with the differentiation process in both groups. However, the hERG channel expression was relatively suppressed in BmKKx2 treated cells (*p*<0.01) (Figure **4B**). The western blotting experiments further confirmed the expression changes of both the up-regulated γ-globin and down-regulated hERG channel in presence of toxin BmKKx2 (Figure **4C**). These data showed that the blockage of toxin BmKKx2 could relatively suppress the hERG channel expression while enhancing K562 cell differentiation.

### BmKKx2 enhancing the differentiation-dependent apoptosis of K562 cells during the erythroid differentiation

As we described before BmKKx2 could decrease the proliferation of K562 cells and cause G1 phase arrest on cell cycle, showing its potential use for leukemia treatment. However, this reduction of cell proliferation did not directly drive cell death. Cell differentiation often accompany with apoptosis, and previous studies showed that the leukemic cells tended to be more sensitive to apoptosis inducers during the differentiation process [25].

**Figure 5:**
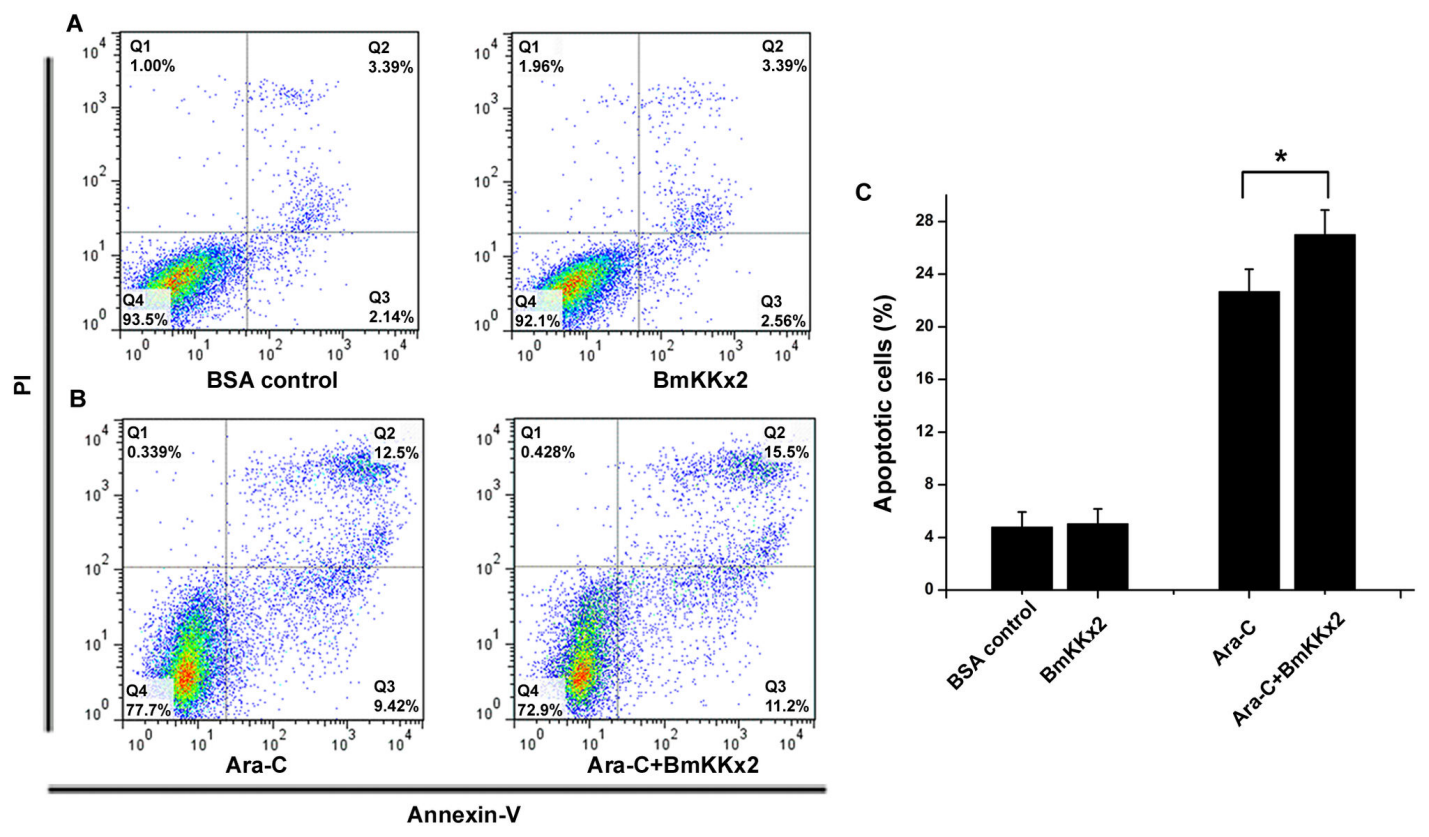
Induction of apoptosis by BmKKx2 during the erythroid differentiation of K562 cells. (A-B) K562 cells were stained with annexin V-FITC and PI and analyzed by flow cytometry. K562 cells were treated with BmKKx2, and BSA was used as the control for 48 h (A). K562 cells were treated with Ara-C (1 μM) and Ara-C (1 μM)+ BmKKx2 for 48 h (B). Flow cytometry data show representative results from one of three independent experiments. (C) Apoptotic cells were stained with annexin V-FITC and PI analyzed by flow cytometry. Values were mean ± SD from three experiments. *p<0.05 (Student’s *t*-test).

Here, we performed the Annexin-V/PI double stain to test the cell death of K562 cells under the different situation. When K562 cells were treated by BmKKx2 alone for 48 h, the ratio of the apoptotic cells showed no difference with BSA control group (Figure **5A**). For K562 cells treated by Ara-C alone for 48 h, a certain extent of apoptosis was detectable, meanwhile the BmKKx2 and Ara-C co-treated group showed an obvious increase on both early apoptosis (from ~9.4% to ~11.2%) and late apoptosis (from ~12.5% to ~15.5%) (Figure **5B and 5C**). These results suggested that differentiated K562 cells were easy to apoptosis due to the blockage of hERG channels.

### Blockage of hERG channel by BmKKx2 causing the Ca^2+^ concentration decrease during the erythroid differentiation of K562 cells

Calcium influx and calcium-dependent proteins play important roles in the erythropoiesis [26,27]. The blockage of hERG channel by toxin BmKKx2 might affect the erythroid differentiation of K562 cells by decreasing the calcium influx.

**Figure 6:**
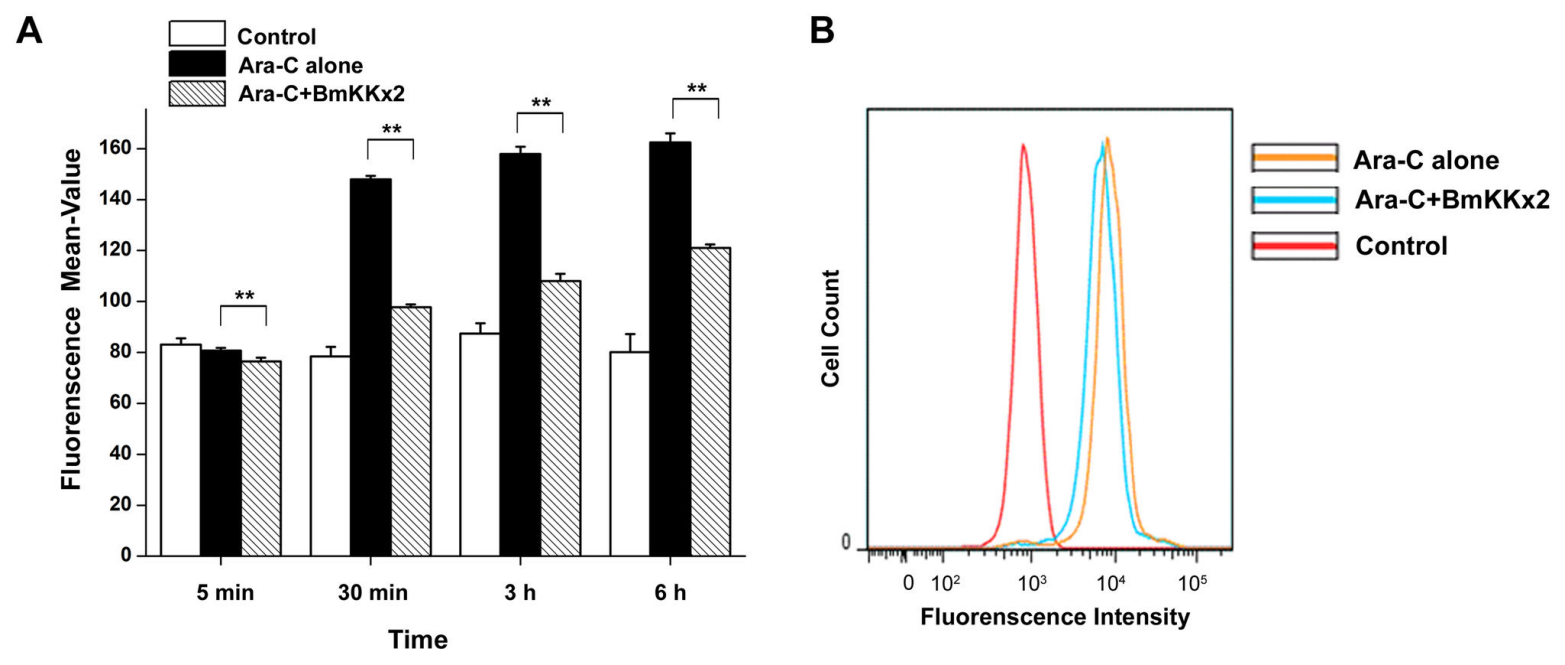
BmKKx2 binding causing the Ca^2+^ concentration decrease during the erythroid differentiation of K562 cells. (A) Intracellular Ca^2+^ was stained by Fluo-8, and the Ca^2+^ concentration was measured by flow cytometric analysis. The mean values were mean ± SD from three independent experiments. **p<0.01 (Student’s *t*-test). (B) Flow cytometric analysis of Fluo-8 fluorescence intensity in K562 cells with or without BmKKx2 after Ara-C induced for 24 h.

In this work, the intracellular calcium concentration ([Ca^2+^]_*i*_) was tested by the flow Cytometry with Fluo-8/AM [28]. As shown in Figure **6A**, [Ca^2+^]_*i*_ in the Ara-C induced K562 cells was dramatically increased from 5 min to 6 h while the [Ca^2+^]_*i*_ in the control group of K562 cells was relatively stable. However, calcium influx driven by the Ara-C was significantly suppressed in the presence of 200 nM BmKKx2 (*p*<0.01) (Figure **6A**). This [Ca^2+^]_*i*_ reduction was still detectable after the cell differentiation continued for 24 h (Figure **6B**). In summary, BmKKx2 could reduce intracellular calcium influx through blocking the hERG channel currents during the erythroid differentiation of K562 cells.

## Discussion

At present, many proteins have been found to affect the erythropoiesis and differentiation of leukemic cells [29-32]. However, the potassium channels, as the diverse and ubiquitous membrane proteins with about one hundred members, are almost neglected in the differentiation of leukemic cells. Significantly, the hERG potassium channel overexpresses in the different leukemic cells, and can modulate the proliferation of the leukemic cells [8,9]. Here, the novel role of hERG potassium channel in the erythroid differentiation of human leukemia cells was investigated.

By using the CML cell line K562 with high hERG channel expression [8,9], the blockage of hERG channel by a potent scorpion toxin BmKKx2 blocker was found to enhance the erythroid differentiation. This effect was further accelerated when K562 cells were pretreated by toxin BmKKx2 (Figures **1** and **2**). This novel function of hERG potassium channel was also confirmed by its knockdown, which resulted into the uninfluence of toxin BmKKx2 on the K562 cell differentiation (Figure **3**). Interestingly, it was also found that the expression of hERG channel was up-regulated during the K562 cell differentiation, and relatively suppressed in the presence of toxin BmKKx2 during the differentiation process (Figure **4**). Previously, the expression of Kv1.3 potassium channels, the target of T cell-mediated autoimmune diseases, was decreased by scorpion toxin ADWX-1 blocker in CD4^+^CCR7^-^ T cells overexpressing Kv1.3 channels [6]. Such common phenomena would be an interesting subject for future study. Together, these similarities of function and expression between the hERG and Kv1.3 channels while interacting with their specific scorpion toxin blockers indicated that hERG channel would be a potential target for developing the novel anti-leukemic drugs to promoting the differentiation of the leukemic cells. 

Calcium, as the most important second messenger, regulates most of cell activities including proliferation and differentiation. Simultaneously, the ionic balance of calcium ion influx and potassium ion efflux is usually critical for the cell activities. In this work, the inhibition of hERG channel currents by scorpion toxin BmKKx2 was found to decrease the intracellular Ca^2+^ concentration during the differentiation process of K562 cells (Figure **6**). This mechanism was also observed in the stimulated CD4^+^ CCR7^-^ T cells whose Kv1.3 channels were blocked by scorpion toxin ADWX-1. Also, ADWX-1 peptide was found to inhibit CD4^+^CCR7^-^ T cell activation through a Kv1.3-mediated IL-2 activation pathway [6]. In the acute myeloid leukemia, the macromolecular signaling complex formed by the hERG channel with VEGFR-1 (FLT-1) and β1 integrin was found to mediate the FLT-1-dependent cell migration and invasion [33]. These studies would be the clues to further explore the mechanism of hERG channel responsible for the enhanced K562 cell differentiation in the future.

 Cell differentiation is always accompanied with apoptosis which is defined as the differentiation-dependent apoptosis. In line with previous findings that hERG channel blockage by E-4031 did not affect the K562 apoptosis [34], our work showed scorpion toxin BmKKx2 could not cause the apoptosis by itself. However, BmKKx2 was able to enhance apoptosis of K562 cells upon Ara-C induction (Figure **5**). Therefore, the differentiation-dependent apoptosis of K562 cells mediated by the hERG channel provided another possible strategy for differentiation therapy.

In addition, scorpion toxins are known peptide blockers interacting with the extracellular pore entryway of the different potassium channels, whose inhibitory mechanism is different from that of the chemical molecule blockers. For example, the similar toxin BeKm-1 blocker was revealed to recognize the extracellular pore entryway of hERG channel (Figure 1A) [11]. However, the residues in the central cavity under the selectivity filter of hERG channel were found to be responsible for the affinity of E-4031 blocker [35]. These different binding mechanisms usually resulted into better selectivity and drug potential for scorpion toxins than chemical molecules nowadays [6,36-38]. For the hERG potassium channel, many specific scorpion toxin blockers were found in recent years [39]. Therefore, the importance of hERG channel in the leukemic pathology would promote the development of scorpion toxins as the prospective drugs. 

In conclusion, we highlighted the important role of hERG potassium channel in the erythroid differentiation of K562 cells. Simultaneously, its specific blocker of scorpion toxin BmKKx2 was found to be able to enhance the erythroid differentiation of K562 cells, which offered a potential therapy via the blockge of hERG channel currents. Due to the overexpression of hERG potassium channels in almost all subcategories of leukemic cells, our findings would accelerate the functional research of hERG channel in the different leukemic cells, and present the prospects of natural scorpion toxins as new anti-leukemic drugs.

## Supporting Information

Figure S1 **Dose-dependence curve of BmKKx2 on erythroid differentiation enhancement of K562 cells.** K562 cells were induced by Ara-C for 48 h under different concentrations of BmKKx2. GPA expression was measured by flow cytometry and the mean values of fluorescence were normalized according to the percentage of max. Symbols and associated error bars represent mean ± SD from three independent experiments in each condition.
(TIF)

Figure S2 **BmKKx2 enhancing the hemin induced erythroid differentiation of K562 cells.** (A) Flow cytometric analysis of GPA fluorescence in K562 cells. K562 cells were treated by hemin for 96 h in the presence or the absence of BmKKx2. BSA control was used to indicate the background expression of GPA. PE-conjugated IgG served as a negative control. (B) Mean fluorescence intensity of GPA shown as mean ± SD from three independent experiments. *p<0.05 (Student’s *t*-test).
(TIF)
