# Supplementary figures and images for: hERG Potassium Channel Blockage by Scorpion Toxin BmKKx2 Enhances Erythroid Differentiation of Human Leukemia Cells K562
Source: PLoS One. 2013 Dec 26;8(12):e84903. doi: 10.1371/journal.pone.0084903 (PMC3873423; doi:10.1371/journal.pone.0084903)

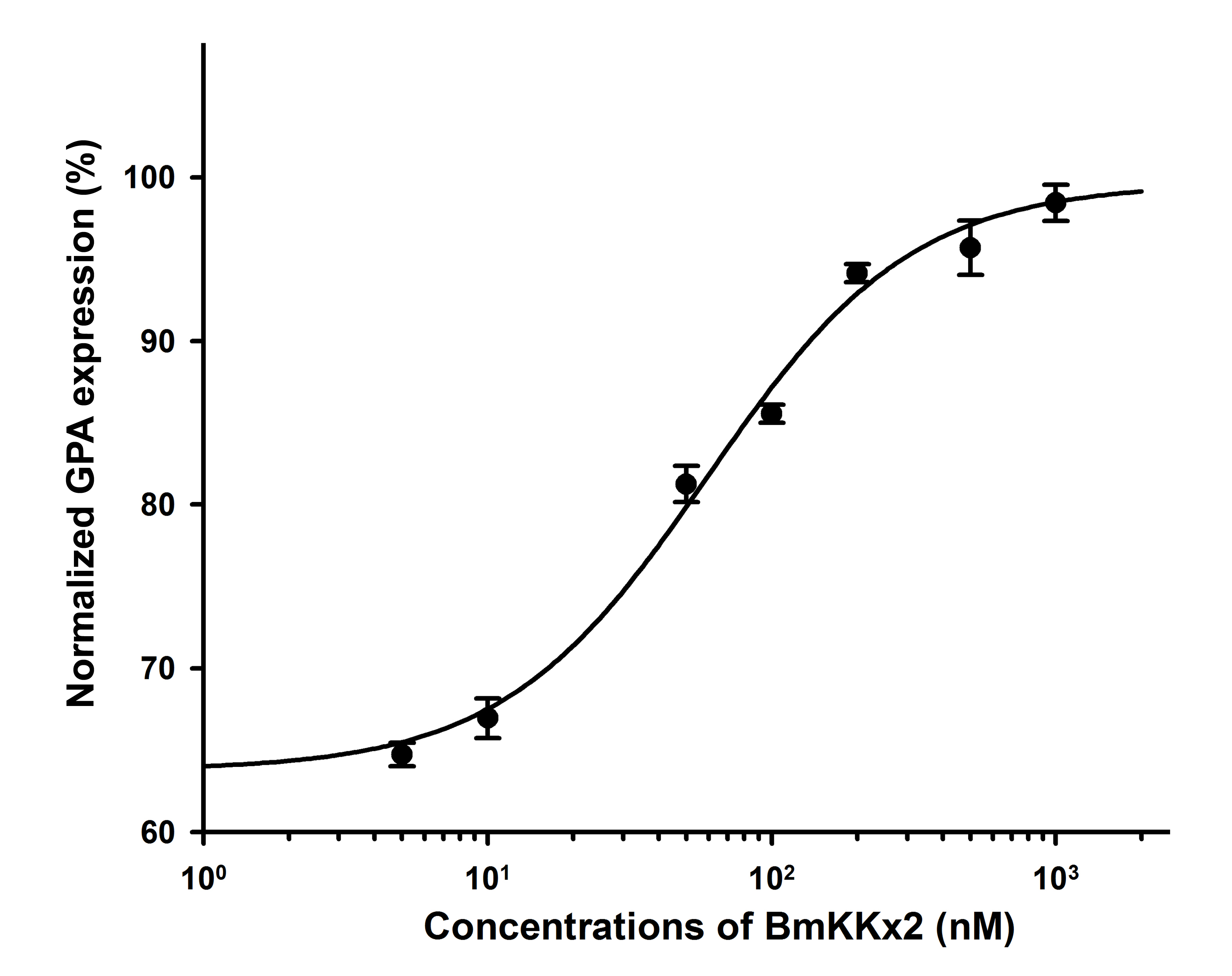

Supplement: Figure S1 — Dose-dependence curve of BmKKx2 on erythroid differentiation enhancement of K562 cells. K562 cells were induced by Ara-C for 48 h under different concentrations of BmKKx2. GPA expression was measured by flow cytometry and the mean values of fluorescence were normalized according to the percentage of max. Symbols and associated error bars represent mean ± SD from three independent experiments in each condition. (TIF) [file pone.0084903.s001.tif]

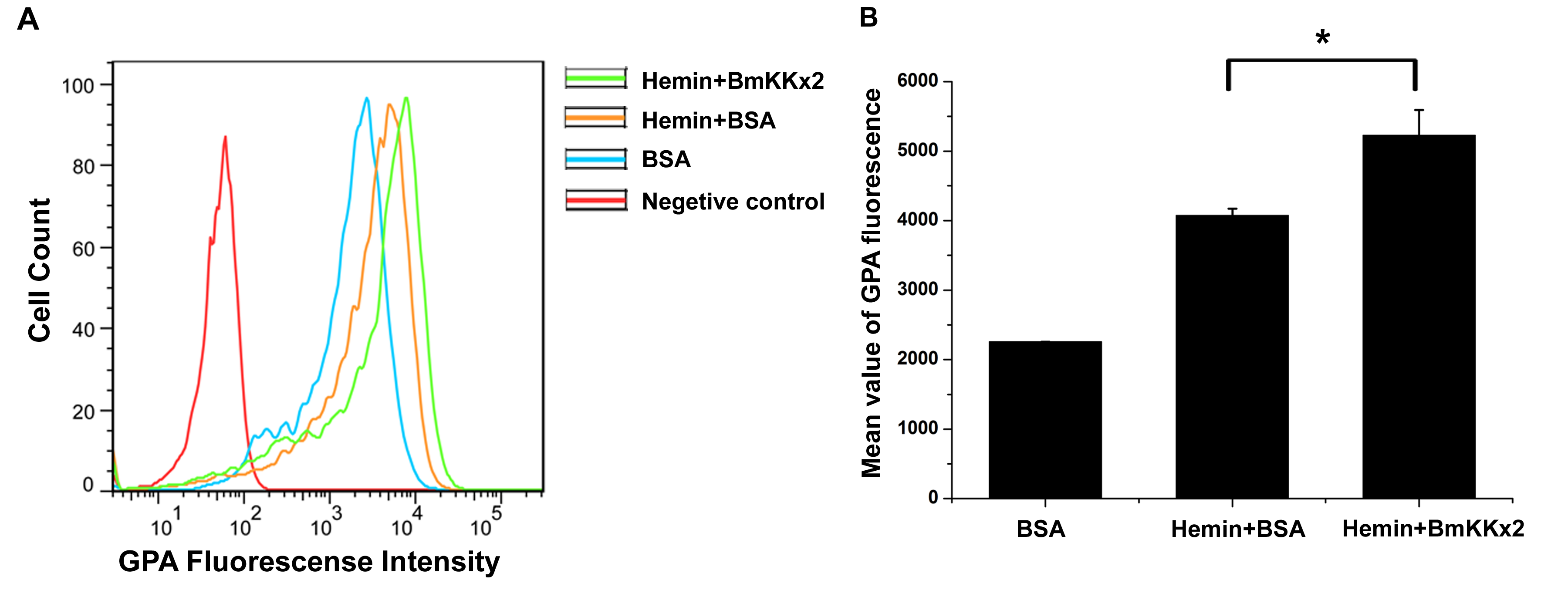

Supplement: Figure S2 — BmKKx2 enhancing the hemin induced erythroid differentiation of K562 cells. (A) Flow cytometric analysis of GPA fluorescence in K562 cells. K562 cells were treated by hemin for 96 h in the presence or the absence of BmKKx2. BSA control was used to indicate the background expression of GPA. PE-conjugated IgG served as a negative control. (B) Mean fluorescence intensity of GPA shown as mean ± SD from three independent experiments. *p<0.05 (Student’s t-test). (TIF) [file pone.0084903.s002.tif]
